# BAFusion: Bidirectional Attention Fusion for 3D Object Detection Based on LiDAR and Camera

**DOI:** 10.3390/s24144718

**Published:** 2024-07-20

**Authors:** Min Liu, Yuanjun Jia, Youhao Lyu, Qi Dong, Yanyu Yang

**Affiliations:** 1 Institute of Advanced Technology, University of Science and Technology of China, Hefei 230088, China; liumin22@mail.ustc.edu.cn (M.L.); lvyouhao@mail.ustc.edu.cn (Y.L.); 2 China Academy of Electronics and Information Technology, Beijing 100041, China; kobej@mail.ustc.edu.cn (Y.J.); yangyanyu@cetc.com.cn (Y.Y.)

**Keywords:** 3D object detection, LiDAR–camera fusion, cross attention

## Abstract

3D object detection is a challenging and promising task for autonomous driving and robotics, benefiting significantly from multi-sensor fusion, such as LiDAR and cameras. Conventional methods for sensor fusion rely on a projection matrix to align the features from LiDAR and cameras. However, these methods often suffer from inadequate flexibility and robustness, leading to lower alignment accuracy under complex environmental conditions. Addressing these challenges, in this paper, we propose a novel Bidirectional Attention Fusion module, named BAFusion, which effectively fuses the information from LiDAR and cameras using cross-attention. Unlike the conventional methods, our BAFusion module can adaptively learn the cross-modal attention weights, making the approach more flexible and robust. Moreover, drawing inspiration from advanced attention optimization techniques in 2D vision, we developed the Cross Focused Linear Attention Fusion Layer (CFLAF Layer) and integrated it into our BAFusion pipeline. This layer optimizes the computational complexity of attention mechanisms and facilitates advanced interactions between image and point cloud data, showcasing a novel approach to addressing the challenges of cross-modal attention calculations. We evaluated our method on the KITTI dataset using various baseline networks, such as PointPillars, SECOND, and Part-A^2^, and demonstrated consistent improvements in 3D object detection performance over these baselines, especially for smaller objects like cyclists and pedestrians. Our approach achieves competitive results on the KITTI benchmark.

## 1. Introduction

Three-dimensional object detection is a critical and challenging task in many applications, such as autonomous driving and robotics [1,2], which require an accurate and robust perception of the surrounding environment and objects in various scenarios. The integration of multiple sensors enhances the accuracy and robustness of 3D object detection by addressing the limitations inherent in single-modality data [3]. Consequently, many methods based on multi-modal fusion have been proposed in recent years.

**Figure 1:**
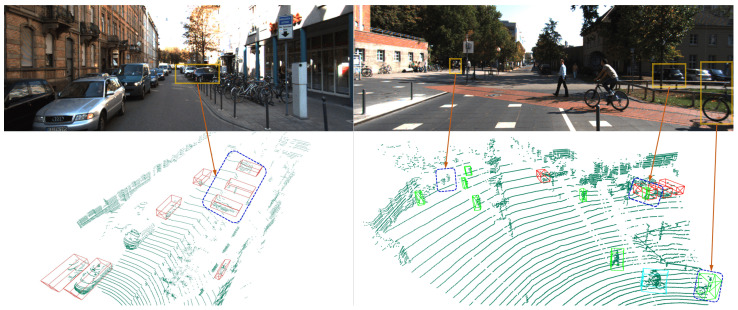
The visualization results of 3D object detection in point cloud space, along with the corresponding images. In the two images on the left, the frequent overlap of objects from the 2D mages from the camera leads to obscuring and thus makes identification difficult. The two images on the right illustrate that due to the absence of visual information such as color and texture, false positives and missed detections are more likely for small targets like pedestrians and cyclists.

Among sensors for 3D object detection, LiDAR and cameras stand out due to their complementary strengths: LiDAR provides precise geometric details through point clouds, while cameras capture rich texture and color information from images. This complementary nature underscores the potential of LiDAR–Camera fusion to enhance the accuracy and robustness of 3D object detection systems [4,5]. Figure 1 illustrates the challenges associated with object overlap and the absence of visual information, highlighting the potential of multi-sensor fusion. Despite this potential, achieving effective fusion poses significant challenges, as it requires aligning and integrating heterogeneous data from these modalities, which differ in characteristics, resolutions, and coordinate systems [6]. The quest for efficient and accurate LiDAR–Camera fusion remains a critical research challenge.

Some early fusion methods for LiDAR–Camera integration, such as projecting LiDAR point clouds onto image planes or generating pseudo-data [7,8,9], provide a simple fusion paradigm but risk information loss and noise from misalignment and scale discrepancies. Conversely, recent approaches, such as leveraging deep neural networks to extract and fuse features at a deep level [10,11,12,13,14], and particularly those utilizing Transformer [15] architectures [16,17,18,19], have markedly enhanced deep feature fusion through the cross-attention mechanism. This mechanism facilitates comprehensive interaction across modalities without necessitating hard alignment, showcasing improved flexibility and the capability for end-to-end training and inference. However, the substantial computational and memory demands of Transformer-based solutions challenge their applicability in real-time scenarios critical for robotics and autonomous driving applications.

Additionally, late-fusion strategies [20,21] combine pre-trained 2D and 3D detectors’ outcomes without retraining, offering flexibility but not addressing the core challenge of multi-sensor data fusion.

In this study, we propose a novel method for multi-modal 3D object detection based on LiDAR and camera fusion, which uses a Bidirectional Attention fusion module to achieve effective and efficient feature fusion. Our method is inspired by the cross-attention from Transformer, which has shown great success in natural language processing and computer vision. However, unlike the existing methods that use Transformer for deep feature fusion, our method does not require heavy computation and memory resources.

The main idea of our method is to design a bi-attention fusion module, called Bidirectional Attention Fusion (BAFusion), which allows the point cloud feature and image feature to interact fully without a projection matrix. The point cloud and image are first fed to their backbones to extract the point cloud features and image features, respectively. Then, the point cloud feature and image feature are input as query–key–value, respectively, to the BAFusion module composed of two cross-attention fusion layers. In each cross-attention fusion layer, features from one modality calculate the attention scores for the other modality. This process adaptively selects and integrates beneficial features, achieving flexible and effective automatic alignment for full multi-modal interaction. Afterward, the features after the cross-attention layer will be concatenated with the features of the other modality and then a convolution layer will be used to further fusion.

To further mitigate the high computational costs typically associated with attention mechanisms in computer vision, we introduce the Cross Focused Linear Attention Fusion (CFLAF) Layer. Drawing inspiration from 2D vision, this layer implements the Focused Linear Attention (FLA) [22] principle to reduce computational demands while enhancing real-time performance significantly. Through the CFLAF Layer, we demonstrate a novel application of focused linear attention within our fusion framework, achieving substantial reductions in time complexity and markedly improving the synergy between LiDAR and camera data for more accurate and efficient 3D object detection.

We evaluate our method on the KITTI [23] dataset, which is a widely used benchmark for 3D object detection. We use different baseline networks, such as PointPillars [24], SECOND [25], and Part-A^2^ [26], and demonstrate that our method can consistently improve the 3D object detection performance over these baselines. Our method achieves competitive results on the KITTI benchmark, and outperforms the classic fusion-based methods.

The main contributions of this paper are summarized as follows:

- A novel method for multi-modal fusion, named BAFusion, a Bidirectional Attention fusion module, is proposed. This module effectively fuses point cloud and image features without hard alignment, ensuring full and bidirectional interaction among cross-modal features. Designed as a plug-and-play component, the BAFusion module can be easily integrated into existing single-modality 3D object detection networks without altering their core structures.
- We adapt and extend the Focused Linear Attention (FLA) [22] into the multi-modal fusion domain with our novel Cross Focused Linear Attention Fusion Layer (CFLAF Layer), markedly reducing computational and memory demands.
- The BAFusion method, incorporating the CFLAF Layer, was evaluated across various baseline networks, including PointPillars, SECOND, and Part-A^2^. It consistently improved 3D object detection performance beyond these baselines and achieved competitive results on the KITTI benchmark.

## 2. Related Work

### 2.1. 3D Object Detection Based on Camera

Over the past decade, camera-based methods have achieved tremendous success in the field of 2D computer vision, leading many researchers to explore their application in 3D object detection. Conventional camera-based approaches typically rely on either monocular or stereo images. Mono3D [27] is among the first to attempt direct regression of an object’s 3D information from a single image, highlighting the potential of monocular images in 3D space understanding.

Similarly, Stereo R-CNN [28] leverages information from multiple camera viewpoints to enhance depth perception. It takes a unique approach by simultaneously processing two stereo images, combining 2D bounding boxes from both left and right images to coarsely compute 3D object boxes, and then refines them using a stereo Region Proposal Network (RPN).

Another category of camera-based methods, which benefits from the advancements in Transformer architectures [15], projects multi-view images into a Bird’s Eye View (BEV) perspective using a view transformer. BEVDet [29] effectively constructs 3D object detectors based on LSS [30]. Additionally, BEVFormer [31] and PETRv2 [32] incorporate temporal information, further enhancing performance through spatio-temporal interactions.

### 2.2. 3D Object Detection Based on LiDAR

LiDAR-based methods are the most popular methods in 3D object detection, and can be divided into point-based, voxel-based, and hybrid point–voxel methods. PointNet [33] and PointNet++ [34] pioneered 3D point cloud understanding by directly processing unordered point clouds using end-to-end neural networks. This innovation significantly benefited subsequent point-based networks in 3D object detection. For instance, PointRCNN [35] utilizes a two-stage network to generate high-quality 3D bounding boxes, while Part-A^2^ [26] leverages free supervision from 3D ground truth boxes to predict high-quality 3D proposals.

On the other hand, voxel-based methods like VoxelNet [36] transform the unstructured point cloud data into regular 3D grids, which facilitates the application of conventional Convolutional Neural Networks (CNNs). This transformation process allows for the efficient processing of point cloud data. Following VoxelNet, SECOND [25] introduced 3D sparse convolution to accelerate 3D voxel processing, significantly improving the efficiency of voxel-based methods. Our BAFusion module can be seamlessly integrated into both point-based and voxel-based methods, demonstrating its versatility and adaptability in enhancing 3D object detection.

Additionally, hybrid point-voxel methods leverage the complementary strengths of both point and voxel approaches. PV-RCNN [37] enhances detection performance by combining voxel-based CNNs with point-based set abstraction, enriching the contextual information for accurate object detection. Meanwhile, 3ONet [38] addresses the challenge of detecting partially occluded objects under obstructed conditions by integrating point segmentation networks with voxel-based reconstruction techniques, demonstrating significant improvements in handling occlusions.

### 2.3. 3D Object Detection Based on LiDAR–Camera Fusion

Recently, there has been a trend to improve the performance of 3D object detection by adopting LiDAR–Camera Fusion [3,4,5], as the LiDAR point cloud and the camera image carry complementary information. AVOD [14] and MV3D [13] are two early works that directly combine 2D and 3D Regions of Interest (RoI). F-PointNet [39] elevates 2D image proposals into the 3D frustum space, conducting further detection within it. PointPainting [7] and PointAugmenting [8] decorate points with semantic scores and features from images. MVXNet [40] performs feature-level fusion by concatenating image and point cloud features. However, all of the above methods require a projection matrix for mapping between 2D and 3D spaces, which may introduce noise due to erroneous mappings. Given its proven capabilities in computer vision, Various LiDAR–Camera fusion methods have been designed based on Transformer. TransFusion [16] generates proposals from LiDAR features and refines them by image-guided object queries and image features key–value. BEVFusion aligns features by converting both point clouds and images into a unified Bird’s Eye View (BEV) perspective. Despite the high potential of Transformer-based fusion methods, the substantial computational cost poses significant challenges for their further application in robotics and autonomous driving fields where real-time performance is required.

## 3. Methods

In this paper, we propose BAFusion, a novel method for 3D object detection based on LiDAR–Camera fusion. By extracting point cloud and image features separately and feeding them into bidirectional cross-attention layers, BAFusion enables the information from different modalities to be softly aligned through a learnable attention score matrix, without hard alignment using a projection matrix. Moreover, to avoid introducing too much computational overhead, we introduce Focused Linear Attention (FLA) [22] to optimize our attention computation, which can significantly reduce the computational cost and improve the speed compared to the conventional Transformer attention.

**Figure 2:**
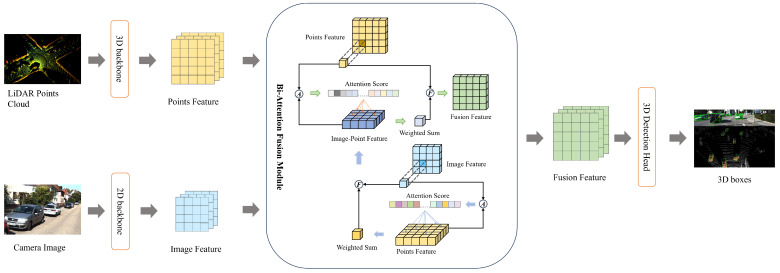
Overview of the BAFusion pipeline. The process starts with separate 3D and 2D backbone networks processing LiDAR point clouds and camera images, respectively. Cross-attention scores are calculated (indicated by *Ⓐ*) to align and fuse the multi-modal features. Our novel fusion process (indicated by *Ⓕ*), detailed in Section 3, further processes the combined features, culminating in the 3D Detection Head, which outputs the detected objects in the scene.

Figure 2 provides a brief overview of the pipeline on how BAFusion accomplishes LiDAR–Camera fusion. In this section, we will highlight the innovative design and operational principles of our method, demonstrating how these layers synergistically enhance the fusion process between LiDAR and camera data. In Section 3.1, we will briefly review the conventional Transformer attention mechanism and introduce the specific computation method of FLA, and we will elucidate the process of utilizing FLA to construct the Cross Focused Linear Attention Fusion Layer. Then, in Section 3.2, we will discuss the specific architecture of BAFusion, which is meticulously constructed using two layers of bidirectional CFLAF Layers.

### 3.1. Cross Focused Linear Attention Fusion Layer

#### 3.1.1. Review of Conventional Transformer Attention

We first revisit the general form of self-attention in conventional Transformer [15]. Transformer is a neural network architecture that relies on the attention mechanism to encode and decode sequential data, such as natural language, speech, or a sequence of vectors. The attention mechanism computes the relevance between each pair of elements in a sequence and uses the weighted sum of the elements as the output. In this way, the attention mechanism can capture both the local and global dependencies in the sequence.

The most common form of attention in Transformer is the multi-head self-attention, which consists of multiple parallel attention heads that operate on the same input sequence. Each attention head first projects the input sequence into query, key, and value vectors using linear transformations, then computes the scaled dot-product attention, for example, given an input sequence $X=\{x_{1},x_{2},\ldots ,x_{n}\}$, where $x_{i}\in R^{d_{x}}$ is the feature vector of the *i*-th element, the scaled dot-product attention is computed as follows:

(1) $$\begin{array}{c} Q=XW_{Q},K=XW_{K},V=XW_{V},\\ \mathrm{Attention}\left(Q,K,V\right)=\mathrm{softmax}\left(\frac{QK^{T}}{\sqrt{d_{k}}}\right)V \end{array}$$

where *Q*, *K*, and *V* are the sequences of query, key, and value vectors, respectively; $W_{Q},W_{K},W_{V}\in R^{C\times C}$ are linear projection weights. $d_{x}$ and $d_{k}$ are the dimensions of the feature vector and key vectors. In this equation, the query and key compute a dot product, which is then passed through a softmax function to generate attention weights. These weights are used to select the parts of the value that the query is most interested in.

Transformer has already been successfully applied to various computer vision tasks, such as image classification [41], object detection [42,43], and semantic segmentation [44]. There are two main methods for feeding images into the Transformer attention. The first method, used by Vision Transformers (ViT) [41], involves dividing the image into patches, and the other one is flattening the image feature, used by DETR [43].

#### 3.1.2. Introduction to Focused Linear Attention

While Transformers have seen significant success in computer vision, they are limited by the $O(N^{2})$ computational complexity. This complexity arises from the softmax-based matrix multiplication used to calculate attention scores between query and key vectors, where *N* represents the length of these vector sequences. This sequence length is far higher than that in NLP, which poses a great challenge for the further development of Transformer attention in computer vision.

**Figure 3:**
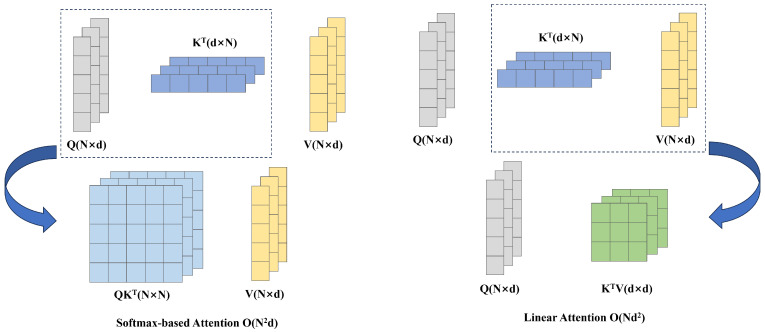
Comparison of softmax attention and linear attention. The matrices Q,K,V denote queries, keys, and values, respectively, with dimensions N×d. This visualization compares the computational complexities of Softmax and linear attention mechanisms, highlighting the efficiency of linear attention, which reduces complexity to $O(Nd^{2})$ by prioritizing the computation of $K^{T}V$ first.

Linear attention [45] is an effective solution that restricts the computation complexity from $O(N^{2})$ to O(N), as shown in Figure 3. By replacing softmax with linear calculation, the self-attention calculation can be expressed as:

(2) $$LA\left(Q_{i},K_{j},V_{j}\right)=\sum_{j=1}^{N}\frac{\phi \left(Q_{i}\right)\phi {\left(K_{j}\right)}^{T}}{\sum_{j=1}^{N}\phi \left(Q_{i}\right)\phi {\left(K_{j}\right)}^{T}}V_{j}$$

where ϕ is a linear function. In this way, we can change the computation order from $(QK^{T})V$ to $Q(K^{T}V)$ based on the associative property of matrix multiplication:

(3) $$LA\left(Q_{i},K_{j},V_{j}\right)=\frac{\phi \left(Q_{i}\right)\left(\sum_{j=1}^{N}\phi {\left(K_{j}\right)}^{T}V_{j}\right)}{\phi \left(Q_{i}\right)\left(\sum_{j=1}^{N}\phi {\left(K_{j}\right)}^{T}\right)}$$

However, simply replacing Softmax attention with linear attention would result in severe performance degradation [46], as the simple linear computation cannot fully capture the importance and diversity of the features as Softmax does. To solve this problem, Focused Linear Attention [22] enhances the focus ability of linear attention by using a carefully designed Focused Function $f_{p}$, and restores the feature diversity by using a depthwise separable convolution layer. Specifically, Focused Linear Attention can be described as follows:

(4) $$FLA\left(Q,K,V\right)=\phi_{p}\left(Q\right)\phi_{p}{\left(K\right)}^{T}V+\mathrm{DWC}\left(V\right)$$

where $\phi_{p}$ is the focused linear function

(5) $$\begin{array}{c} \phi_{p}\left(x\right)=f_{p}\left(\mathrm{ReLU}\left(X\right)\right),\\ f_{p}\left(x\right)=\frac{∥x∥}{∥x^{**p}∥}x^{**p} \end{array}$$

where $x^{**p}$ represents element-wise power p of x. By leveraging the Focused Function to recalibrate the orientation of query and key vectors, FLA can enhance the attention between query-key pairs with stronger relevance, while reducing the attention between those with weaker relevance. In summary, Focused Linear Attention maintains low computational complexity while preserving high expressive power, making it well-suited for multi-modal 3D object detection tasks.

#### 3.1.3. Architecture of CFLAF Layer

**Figure 4:**
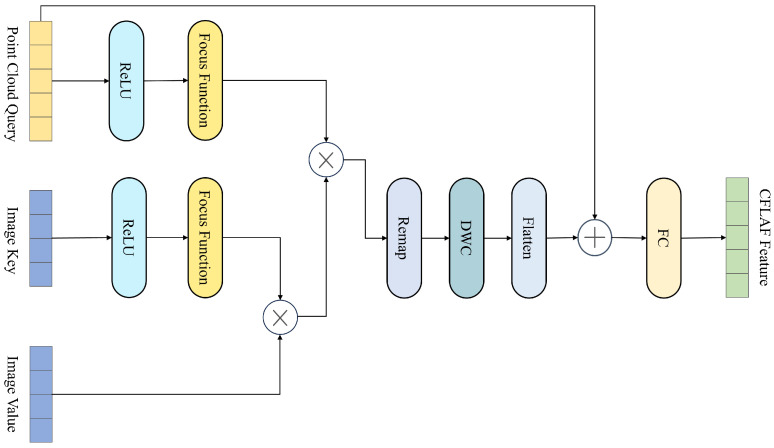
Schematic of the CFLAF Layer structure. This diagram illustrates the specific structure and workflow of the Point-Image CFLAF Layer. The input includes point cloud query from the points feature and image key–value from the image feature. The symbol ⨂ represents matrix multiplication, and ⨁ represents element-wise addition. Note that different from the self-attention in the 2D image domain, the source and the sequence length of query and key–value can be different in CFLAF Layers. The attention output is passed through a fully connected layer and added to the query to achieve preliminary fusion.

In order to better utilize the attention mechanism for LiDAR–Camera fusion, based on FLA, we construct a Cross Focused Linear Attention Fusion Layer (CFLAF Layer), for implementing the attention interaction and preliminary fusion of LiDAR point cloud and camera image information. The structure of the CFLAF Layer is illustrated in Figure 4, where an example of the Point-Image CFLAF Layer is provided to demonstrate the process of attention interaction and preliminary fusion between LiDAR point cloud and camera image features.

In the CFLAF Layer, a meticulous fusion process is designed to effectively integrate information from point cloud queries and image key–value pairs. The input to this layer consists of point cloud queries can be denoted by $Q\in R^{N_{p}\times d}$ and image key–value pairs can be represented as matrices $K\in R^{N_{i}\times d}$ and $V\in R^{N_{i}\times d}$. Here, $N_{p}$ represents the number of point cloud queries, and $N_{i}$ denotes the number of image key–value pairs, corresponding to positions within the point cloud and image feature spaces, respectively. *d* represents the common feature dimension. While $N_{p}$ and $N_{i}$ may vary, *d* usually remains constant across modalities.

The operation within the CFLAF Layer initiates by performing a matrix multiplication between the value matrix V and the transpose of the key matrix $K^{⊤}$, which yields a time complexity of $O(N_{i}d^{2})$ and results in an intermediate matrix of size d×d. Following this, the d×d intermediate matrix is combined with the point cloud query matrix Q through an additional matrix multiplication, involving the dimensions $N_{p}\times d$ and d×d. This operation contributes an extra time complexity of $O(N_{p}d^{2})$, with $N_{p}$ denoting the number of point cloud queries.

As such, the overall computational complexity of the attention mechanism in the CFLAF Layer can be expressed as $O(\left(N_{i}+N_{q}\right)d^{2})$. Given that in practical applications, $N_{i}$ and $N_{q}$ are significantly larger than the feature dimension d, the CFLAF Layer based on FLA achieves a substantial reduction in computational complexity compared to conventional cross-attention methods that exhibit a complexity of $O(N_{i}N_{q}d)$.

Through this carefully orchestrated sequence of computations, the CFLAF Layer enables the adaptive selection of relevant image features for each point cloud query. The final output is an $N_{q}\times d$ vector, where each entry reflects the weighted sum of image values determined by learnable attention scores assigned to the corresponding image keys. This weighting mechanism empowers the model to assign higher importance to image locations that are beneficial to understanding the current point cloud feature context, while down-weighting or disregarding those that are irrelevant or potentially detrimental.

Ultimately, these attentively selected and weighted image features are element-wise added to the original point cloud queries after passing through a fully connected layer. This operation fosters deep interaction between the two modalities and achieves preliminary fusion at the CFLAF Layer. This design strategy not only maintains high efficiency due to its reduced computational requirements but also ensures that the fused representation captures essential multi-modal information without excessive parameterization.

### 3.2. Bidirectional Attention Fusion Module

The BAFusion module takes point cloud and image data as inputs and feeds them into separate feature extraction networks. The features of the point cloud and image are obtained after passing through the Backbone and Neck layers of each network. The feature maps may have different shapes, while the number of channels should be the same.

Following the method of DETR [43], the image and point cloud features are flattened in the width and height dimensions while keeping the channel dimension unchanged. Specifically, given that the shape of the image feature map is $H_{i}\times W_{i}\times C$ and the shape of the point cloud feature map is $H_{p}\times W_{p}\times C$, after flattening, the shapes of the image and point cloud feature sequences will be $N_{i}\times C$ and $N_{p}\times C$, respectively, where $N_{i}=H_{i}\times W_{i}$ and $N_{p}=H_{p}\times W_{p}$.

**Figure 5:**
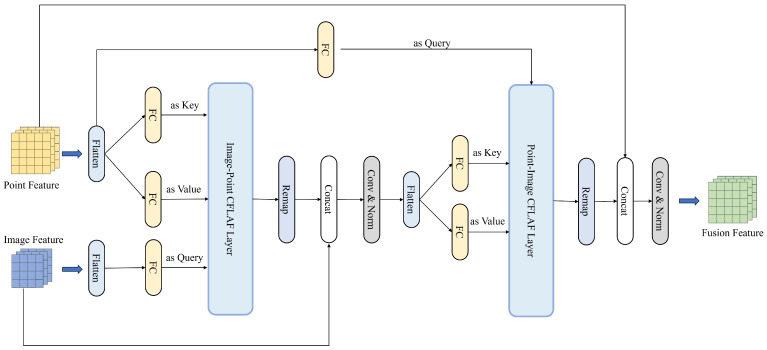
The LiDAR–Camera fusion process of the BAFusion module. It involves Bidirectional Attention layers: Image–Point CFLAF Layers and Point-Image CFLAF Layers. The former adaptively selects and fuses beneficial point cloud information to the image feature, while the latter does the same for image information to the point feature. Channel concatenation and convolution layers further enhance the fusion, resulting in a feature rich in both point cloud and image information.

The BAFusion module initially maps the flattened features to query–key–value through a fully connected layer. Subsequently, a cascade of bidirectional CFLAF Layers is employed to ensure thorough feature interaction and information supplementation, as shown in Figure 5.

Firstly, the flattened point cloud features are transformed into key–value pairs via a fully connected layer, which is then input into the first Image–Point CFLAF Layer. Simultaneously, the image features are mapped to query through another fully connected layer and also fed into the Image–Point CFLAF Layer. Within this layer, the image features, acting as query, engage in cross-attention with the point cloud key. A trainable attention score matrix is utilized to assign larger weights to point cloud values that exhibit stronger associations and provide meaningful supplementary information, while those with weaker associations or less meaningful information receive less attention. This adaptive selection process ensures the Image–Point CFLAF Layer effectively fuses beneficial point cloud information, achieving preliminary fusion.

The output feature from the Image–Point CFLAF Layer, denoted as $F_{IP}$, is remapped back to the original feature map shape. Because the shape of $F_{IP}$ aligns with the input query, the remapped feature map maintains the same shape as the initial image feature. Subsequently, the remapped feature map and the original image feature are concatenated by channel, forming a $H_{i}\times W_{i}\times 2C$ feature map. Through convolution followed by BatchNorm, we achieve further interaction among point cloud and image features, compressing the channel back to *C* and resulting in a feature map $F_{IP^{\prime }}$ representing the fully integrated image feature that has absorbed beneficial information from the point cloud. The first fusion process can be succinctly expressed as:

(6) $$F_{IP^{\prime }}=\mathrm{Conv}\left(\mathrm{Concat}\left(\mathrm{Remap}\left(\mathrm{CFLAF}\left(Q_{i},K_{p},V_{p}\right)\right),F_{i}\right)\right))$$

where $Q_{i}$ denotes the image query, and $K_{p}$ and $V_{p}$ represent the point cloud key and value, respectively. $F_{i}$ denotes the feature map of the image.

After the first fusion process, the obtained feature map $F_{IP^{\prime }}$ is also flattened in the same way as DETR, and then mapped to key–value through a fully connected layer. This mapped feature sequence is then input into the Point-Image CFLAF Layer, where it serves as key–value, while the original point cloud feature sequence is employed as query through another fully connected layer. In the second Point-Image CFLAF Layer, cross-attention occurs between the point cloud query and the image key. Utilizing a learnable attention score matrix, adaptive selection of beneficial image information is achieved, similar to the process in the Image–Point CFLAF Layer. The image value with larger association and beneficial information is assigned a larger weight, while those image values with less association or harmful information are given less attention.

The feature output from the Point-Image CFLAF Layer can be represented as $F_{PI}$, and $F_{PI}$ can also be remapped back to a feature map with the same shape as the initial point cloud feature, and then concatenated with the initial point cloud feature by channel to generate a $H_{p}\times W_{p}\times 2C$ feature map. Through subsequent convolution and BatchNorm operations, we achieve deeper point cloud-image information interaction, and a final fusion feature of $H_{p}\times W_{p}\times C$ is generated. This feature, which contains rich and complementary point cloud image information through the cascade of bidirectional CFLAF Layers, will be input into the next step of the Baseline network as the final fusion feature and achieve the final 3D object detection.

Overall, a complete BAFusion process can be simply expressed as:

(7) $$F_{fusion}=F_{PI}^{\prime }=\mathrm{Conv}\left(\mathrm{Concat}\left(\mathrm{Remap}\left(\mathrm{CFLAF}\left(Q_{p},K_{ip},V_{ip}\right)\right),F_{p}\right)\right)$$

where $Q_{p}$ denotes the point cloud query, $K_{ip}$ and $V_{ip}$ denote the key and value from the Image–Point feature, and $F_{p}$ denotes the feature map of the point cloud.

## 4. Experiment

In this section, we present the experiments we conducted to evaluate the performance and effectiveness of our proposed module, BAFusion, for multi-modal 3D object detection.

We first introduce the dataset and metric used for our experiments. Then we describe the implementation details, including the environment of the experiment, the model setting, and the process of training and testing. Next, we report and analyze the experimental results on the KITTI [23] test set and the validation set, and compare them with other methods. Finally, we conduct ablation studies to investigate the impact of different components and parameters.

### 4.1. Dataset and Metric

Our experiments are based on the KITTI [23] open benchmark dataset, which is one of the most used datasets for object detection in autonomous driving, containing 2D, 3D, and bird’s eye view detection tasks. Equipped with LiDAR and cameras, KITTI collected 7481 training images and 7518 test images as well as the corresponding point clouds. Three object types—cars, pedestrians, and cyclists—are labeled, with over 200,000 3D object annotations. These annotations are divided into three categories based on detection difficulty: easy, medium, and hard. Following [13,47], we split the KITTI training set into separate training and validation subsets, each with a comparable number of samples. Specifically, the training set comprises 3712 samples, while the validation set comprises 3769 samples.

For the 3D object detection task on the KITTI dataset, the Average Precision (AP) metric is commonly employed for evaluation and comparison. AP evaluates the performance of object detection models for individual classes. It is computed by constructing a Precision–Recall Curve based on the confidence threshold and calculating the area under the curve. Precision and Recall, reflecting the accuracy and completeness of predicted bounding boxes, are determined by the Intersection over Union (IoU) between predicted and ground truth bounding boxes. IOU can be expressed as

(8) $$IOU=\frac{Area\;of\;Overlap}{Area\;of\;Union}$$

The IOU threshold for determining the correctness of a prediction varies across different classes. In the KITTI dataset, the IOU threshold is set at 0.7 for the car class and at 0.5 for both the cyclist and pedestrian classes. Based on these thresholds, we can then calculate Precision and Recall as follows:

(9) $$Precision=\frac{True\;Positive}{True\;Positive+False\;Positive}$$

(10) $$Recall=\frac{True\;Positive}{True\;Positive+False\;Negative}$$

where True Positive is the number of correctly predicted bounding boxes, False Positive is the number of incorrectly predicted bounding boxes, and False Negative is the number of missed bounding boxes.

### 4.2. Implementation Details

#### 4.2.1. Experiment Environment

All of the experiments are conducted in the following environment:

1. OS: Ubuntu 20.04
2. GPU: Single GeForce RTX 4090 (NVIDIA, Santa Clara, CA, USA)
3. CUDA: 11.3
4. Python: 3.9.18
5. PyTorch: 1.11.0
6. MMDetection3D [48]: 1.4.0

#### 4.2.2. Model Setup

We primarily base our work on existing point cloud baseline networks, including SECOND [25], PointPillars [24], and Part-A^2^ [26]. These networks serve as the main branch of our model. For image feature extraction, we employ a separate branch composed of a Backbone and a Neck. The Backbone mainly employs ResNet [49], while CSPNet [50] from the YOLO [51] series offers a lightweight alternative for the image Backbone. To enhance the multi-scale information of the image features, we further employ a PAN-FPN [51] following the Backbone feature extraction.

Subsequently, the point cloud features and image features are fed into our proposed BAFusion module, which facilitates the fusion of LiDAR–Camera multi-modalities. The fusion features are then sent to the head of the point cloud baseline to achieve multi-modal 3D object detection.

#### 4.2.3. Training Schedule

In this section, we detail the training procedures of our models, which are conducted end-to-end on a single Geforce RTX4090. For the image branch, we load pre-trained weights from ImageNet for ResNet and from COCO for CSPNet.

The learning rates for BA-Second and Part-A^2^ are set to 0.0018 and 0.001, respectively, with AdamW chosen as the optimizer. The learning rate adopts the cyclic schedule, and both are trained for 80 epochs on the KITTI dataset. For BA-PointPillars, the learning rate is set at 0.0005, with the same optimizer and training schedule applied, trained for 160 epochs.

Considering the need to maintain correspondence between images and point clouds in multi-modal tasks, we use a ground truth sample data augmentation method that allows for the simultaneous transformations of both point clouds and images. Similar to the approaches in AutoAlignV2 [17] and PointAugment [8], this method enables sampling various category instances from pairs of corresponding point cloud and image samples, and randomly pasting these instances into other samples. We slightly adjust these methods to better fit the KITTI dataset.

Additionally, we maintain the synchronized flipping data augmentation for point cloud and image. Following the common practice of MVXNet [40] and most point cloud methods, we employ random global rotations and translations to enhance the robustness of our model to spatial transformations. However, we discard other complex point cloud and image data augmentations that could alter the correspondence between images and point clouds. Despite using fewer data augmentations compared to single-modal methods, our method still achieves superior results. This demonstrates the robustness of our approach and its ability to effectively leverage the available data.

### 4.3. Experiment Result

#### 4.3.1. Evaluation on KITTI Test Set

To evaluate the effectiveness of our method, we conducted experiments on the KITTI test set, comparing our approach with the established baseline. Firstly, the performance of three baseline models—PointPillars, SECOND, and Part-A^2^—is examined, both with and without the integration of BAFusion. This evaluation aims to illustrate the improvements BAFusion brings to these models. Subsequently, we compare our enhanced model, BA-Part-A^2^, against other typical methods that have reported their results on the KITTI benchmark, to demonstrate the competitive performance of our model. The BAFusion models evaluated on the KITTI test set all use ResNet as their image backbone.

The results, presented in Table 1 and Table 2, show significant detection performance improvements after integrating the BAFusion module into the baseline networks. After integrating the BAFusion module, we observed improved detection performance across nearly all categories and difficulty levels. This improvement was especially significant for smaller objects, including cyclists and pedestrians. Such objects likely benefit from the additional texture and color information provided by images. This demonstrates the BAFusion method’s effectiveness in achieving cross-modal information fusion.

In addition, both voxel-based methods, such as PointPillars and SECOND, and point-based methods, such as Part-A^2^, can fully incorporate information from images and enhance their performance when using our BAFusion module. This demonstrates the versatility of our BAFusion module.

Moreover, our Part-A^2^ model exhibits competitive, if not superior, performance when compared to other classic methods, underscoring the potential of BAFusion in advancing 3D object detection. As illustrated in Table 3 and Table 4, our BA-Part-A^2^ model demonstrates significant improvements over traditional single-modal point cloud detection networks like PointRCNN [35], as well as classic multi-modal 3D detection frameworks such as PointPainting [7]. Particularly noteworthy is its commendable performance in the Cyclist category.

In conclusion, our evaluation on the KITTI test set highlights the exceptional capability of the BAFusion module in effectively merging image information into point cloud detection networks. By leveraging this efficient fusion mechanism, our approach showcases excellence in multi-modal information fusion and complementarity, significantly enhancing 3D object detection performance. The demonstrated versatility and transferability of the BAFusion module across various baseline networks underscore its plug-and-play nature, further emphasizing its potential for broader adoption in the field.

#### 4.3.2. Evaluation on KITTI Validation Set

As the test annotations of the KITTI dataset are not publicly available, we cannot flexibly evaluate our method on the KITTI test set. However, thanks to the widely adopted partition method [13,47], we can still obtain a fair comparison result on the KITTI validation set.

To elucidate the enhancements afforded by the integration of BAFusion, we present comparative analyses focusing on 3D and BEV performance metrics across our selected point cloud baselines (as shown in Table 5 and Table 6). All models evaluated on the KITTI validation set are trained in the same experimental environment, based on MMDetection3D [48] v1.4.0, ensuring fairness and consistency in evaluation and comparison.

Furthermore, we conducted additional comparisons with some of the state-of-the-art fusion methods recently developed, such as BEVFusion [55] and SupFusion [56]. Although some of these methods do not provide their implementations or results on the KITTI dataset, we successfully reproduced them using MMDetection3D and evaluated their performance on the KITTI validation set. Throughout the evaluation process, we ensured that all experimental environments were consistently maintained. We integrated the SupFusion fusion module into the same baseline as our BAFusion, applying identical learning rates and training strategies. Meanwhile, BEVFusion was trained for 80 epochs with a learning rate of 0.001, following the consistent training schedule. Table 7 and Table 8 present a performance comparison of the baselines integrating the SupFusion fusion module, BEVFusion, and our BAFusion method.

Experimental results indicate that SupFusion performs better in the Car category, thanks to its innovative Polar Sample [56] method, which densifies the point clouds of objects in scenes to enhance feature expressiveness. However, this improvement primarily results from an increase in point cloud data. In contrast, our BAFusion, which employs a Bidirectional Attention module, focuses more on enhancing the complementary information exchange and absorption between different modal features, allowing the network to more effectively utilize the existing rich information of multi-modal data. When evaluating performance in the Pedestrian and Cyclist categories, SupFusion does not exhibit a marked advantage compared to BAFusion, further highlighting the potential and effectiveness of BAFusion in multi-modal fusion. On the other hand, BEVFusion shows certain advantages in BEV performance comparisons, attributed to its BEV-centric feature processing workflow. However, when performing in single-view scenarios such as KITTI, BEVFusion fails to demonstrate the expected definitive advantages, revealing the challenges of constructing effective BEV features directly from single-image data. This observation also inspires us to consider further modifications to the BAFusion module to potentially incorporate information from multi-view image data, which could likely enhance the performance of our method.

### 4.4. Visualization of Detection Results

**Figure 6:**
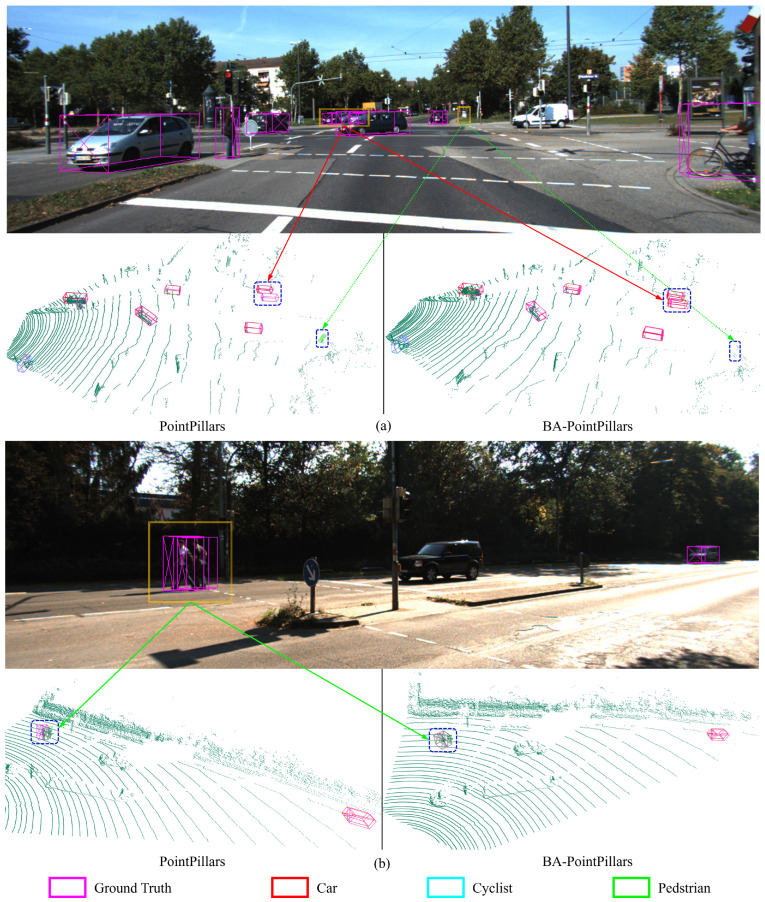
Comparative visualization of detection results between PointPillars and BA-PointPillars. (**a**) Scenario 1 highlights an avoidance of the false-positive detection of a road sign as a pedestrian and the accurate differentiation of closely positioned vehicles by BA-PointPillars, showcasing the model’s enhanced discernment with the aid of visual information. (**b**) Scenario 2 demonstrates BA-PointPillars’ ability to accurately identify two adjacent pedestrians, in contrast to PointPillars’ merged detection.

In our comparative analysis, two distinct samples were meticulously selected to demonstrate the enhanced detection capabilities of BA-PointPillars, as exemplified in Figure 6. This method leverages the synergistic integration of LiDAR and camera data, enabling it to address the challenges associated with LiDAR-only networks and outperform the standard PointPillars approach. The first scenario highlights a critical case where PointPillars conflated two adjacent pedestrians into a single detection due to the inherent limitations of spatial resolution in LiDAR data alone. Conversely, BA-PointPillars, benefiting from the auxiliary visual cues, accurately differentiated and detected both individuals. This instance underscores the augmented perception achieved through the fusion of modalities, effectively mitigating the issue of missed detections inherent to relying solely on point cloud data.

The second sample further exemplifies the superiority of BA-PointPillars in distinguishing between pedestrians and non-pedestrian objects in challenging scenarios. Specifically, PointPillars erroneously identified a distant road sign as a pedestrian, a common error induced by the sparse nature of distant LiDAR scans that render distinct objects indistinguishable. In contrast, BA-PointPillars, with the inclusion of image data, readily discerned the true nature of the road sign, thus avoiding the false positive. Additionally, the fusion approach exhibited remarkable precision in detecting and individually identifying two closely situated vehicles, a task that poses significant challenges in dense traffic scenes.

Through these visualizations, we demonstrate the pivotal role of multi-modal fusion in enhancing the accuracy and reliability of 3D object detection systems.

### 4.5. Ablation Studies

This subsection delves into ablation studies dissecting individual BAFusion module component contributions to 3D object detection performance. Experiments elucidate the effectiveness and efficiency of proposed enhancements over the baseline model.

#### 4.5.1. Experimental Setup

We base our experiments on the KITTI Val set. Evaluation metrics include Average Precision (AP) for object detection accuracy, computational speed, memory consumption, and model size for efficiency assessments. All experiments were conducted in the same environment for fair comparisons. Unless otherwise specified, the baseline network is PointPillars [24], and the BA-model is BA-Pointpillars with the CSPNet [50] as its image backbone.

#### 4.5.2. Fusion Mechanism Ablation

*Sequential vs. Parallel*

**Figure 7:**
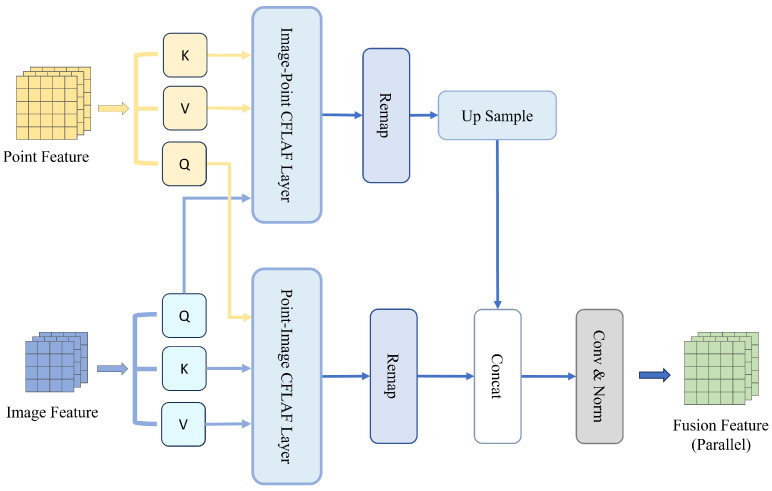
Illustration of the parallel architecture for the BAFusion module. Unlike sequential processing methods, this parallel approach independently computes attention for Point–Image and Image–Point, aligns the feature maps via up-sampling, and then proceeds with subsequent processing steps.

We also designed a parallel fusion pipeline for BAFusion, the structure of which is shown in Figure 7, to justify our chosen approach.

As shown in Table 9, our experimental results indicated a superior performance of the sequential fusion process over the parallel process. This can be attributed to the following two reasons:

**Progressive Fusion and Information Integrity:** In the sequential architecture, the fusion and selection of features from different modalities are executed step-wise. At each step, attention is focused on one modality interaction, allowing for a progressive transfer and refinement of information between modalities. This ensures a consistent feature space where cross-modal features are meticulously aligned and integrated, enhancing the overall accuracy of the detection system. In contrast, parallel processing independently handles two groups of cross-modal attention computations, lacking attentiveness to the other branch. This leads to poor compatibility between the two types of features, potentially resulting in rough and less effective fusion.

**Feature Map Dimension Consistency:** Due to the nature of attention mechanisms, the dimensions of the resulting feature map conform to those of the query input. In sequential processing, this characteristic ensures an uninterrupted flow, where the feature maps are directly compatible with subsequent concatenation and convolution operations without the need for additional manipulation. Conversely, in parallel processing, attention computations are performed simultaneously for both modalities, leading to discrepancies in the resultant feature map dimensions. This discrepancy necessitates an up-sampling step to align the feature maps from different modalities, which introduces two main issues: information loss and blurring due to interpolation, and potential misalignment of features that were originally well-positioned through attention mechanisms. Such misalignments can degrade the effectiveness of the fusion process, hindering the model’s ability to accurately integrate and align multi-modal information.

*Single Attention vs. Bidirectional Attention*

To evaluate the efficacy of the bidirectional layers in our BAFusion module, we conducted an ablation study. This study compared the 3D detection performance of configurations based on Single Attention (SA) and Bidirectional Attention (BA) within the BA-PointPillars framework.

As shown in Table 10, while the SA-based approach does offer an improvement over the baseline, the BA-based model demonstrates better performance across all categories. In particular, the BA-based approach demonstrates substantial improvements in detecting cyclists and pedestrians, which underscores the Bidirectional Attention layers’ superior ability to capture intricate details and process complex scenes comprehensively.

The improved AP of 3D detection, particularly in complex scenarios, validates the Bidirectional Attention layer as a more sophisticated approach to multi-modal fusion.

*FLA vs. Conventional and Linear Attention*

We compare the BAFusion module based on Focused Linear Attention (FLA) [22] against variants employing Conventional Attention (CA) and a typical example of general linear attention, such as Efficient Attention (EA). The CA-based model required training for 240 epochs due to its challenging convergence properties, whereas both the EA-based and FLA-based approaches achieved convergence with only 160 epochs. As shown in Table 11, the FLA-based BA-PointPillars demonstrates comparable or superior performance in 3D object detection accuracy across various categories and difficulty levels when compared to its CA-based counterpart. The performance of the EA-based method, however, was only marginally better than the baseline, likely due to linear attention mechanisms tend to generate smoother attention scores relative to Conventional Attention, which may hinder effective feature capture, as discussed in [46,57]. In contrast, the focus function employed by FLA sharpens the distribution of attention scores, thereby enhancing the weighting of features with stronger relevance. This mechanism enables the more effective capture of inter-modal feature relationships. The experimental results confirm the potential of FLA to integrate multi-modal features more adeptly.

The computational benefits of FLA are made starkly apparent in Table 12. Admittedly, compared to the original baseline, the introduction of additional image branches and the incorporation of attention modules inevitably lead to a decrease in computational efficiency. However, compared to Conventional Attention, FLA demonstrates substantial computational advantages. Specifically, the FLA (BA) model not only doubles the inference speed (26.8 FPS over 13.9 FPS for the CA-based approach) but also attains an over 80% reduction in memory consumption (from 5025 MB to 650 MB) and a significant decrease in model size (from 185 MB to 115 MB). These drastic improvements in inference speed and resource utilization, coupled with the reduced training duration (160 vs. 240 epochs for CA), underscore the practicality of FLA in real-world applications where efficiency is paramount.

The experimental results demonstrate our method’s efficiency and adaptability in processing large multi-modal datasets, like point clouds and images, proving its suitability for various complex tasks in robotics and autonomous driving.

#### 4.5.3. Impact of Image Feature Quality

The image backbone significantly dictates the quality of image feature extraction, which is critical for the fusion process in multi-modal feature fusion. To evaluate the impact of image feature quality, we conduct experiments utilizing CSPNet [50] and ResNet [49]. ResNet, with its deeper and wider architecture, typically yields a more substantial feature map, whereas CSPNet’s design is more lightweight and efficiency-oriented.

Table 13 presents the performance comparison of the two backbone networks, with and without the benefit of pre-trained models. The results indicate that while both architectures benefit from pre-trained models, the extent of improvement varies between them.

In the Cars category, ResNet and CSPNet exhibit similar results, likely due to the larger size of vehicles. This suggests that point cloud information alone may be sufficient for their accurate representation in the feature space, resulting in a reduced dependency on image features. In contrast, detection results for cyclists and pedestrians indicate that the introduction of image features consistently enhances detection performance, regardless of the backbone used or the inclusion of pre-trained weights. This underscores the necessity for additional semantic information in recognizing smaller objects. Overall, ResNet appears to be superior, suggesting that its larger feature maps and more feature channels provide a richer scene representation, thereby enhancing the multi-modal model’s ability to capture detailed semantic information for 3D detection. Notably, with pre-trained weights, the CSPNet-based model shows significant improvement in the Pedestrian category. This improvement may stem from the learned ability to better recognize the Human category, facilitated by pre-training on the COCO dataset. This observation highlights the potential benefits of leveraging pre-trained models from 2D object detection or similar tasks to enhance feature extraction for 3D object detection.

#### 4.5.4. Influence of Position Encoding

The role of position encoding (PE) in attention mechanisms is widely studied across attention-based methods, generally considered essential for providing relative spatial context between feature vectors. We evaluate the effects of different position encoding strategies, specifically fixed sine–cosine, learnable, and the absence of position encoding, on the performance of our model.

The comparative results displayed in Table 14 reveal that omitting position encoding altogether yields the most favorable outcome. This counterintuitive finding can be attributed primarily to our distinctive fusion process, which entails “remap->concat->conv”. This process effectively restores 1D sequence features back to 2D feature maps, inherently preserving the spatial relationships that are vital for understanding the scene geometry.

The convolutional layers subsequently exploit this preserved spatiality to seamlessly integrate local multi-modal information. This integration is pivotal as it leverages the inherent structure of the data, enhancing feature representation without relying on the arbitrary imposition of position embeddings. Conversely, PE like fixed sine–cosine position encoding introduces an extraneous form of spatial awareness that can misalign with the natural spatial structures in LiDAR and image data, thereby injecting noise and potentially degrading model performance.

#### 4.5.5. Attention Visualization

We also delve into the role of cross-attention in the BAfusion module through the lens of attention visualization. We select a random head of the last attention layer and average all queries to show which image information the entire point cloud feature space pays more attention to. By visualizing the attention scores between point cloud queries and image key–value pairs, we provide direct insight into how the BAFusion module autonomously selects relevant image features for fusion.

**Figure 8:**
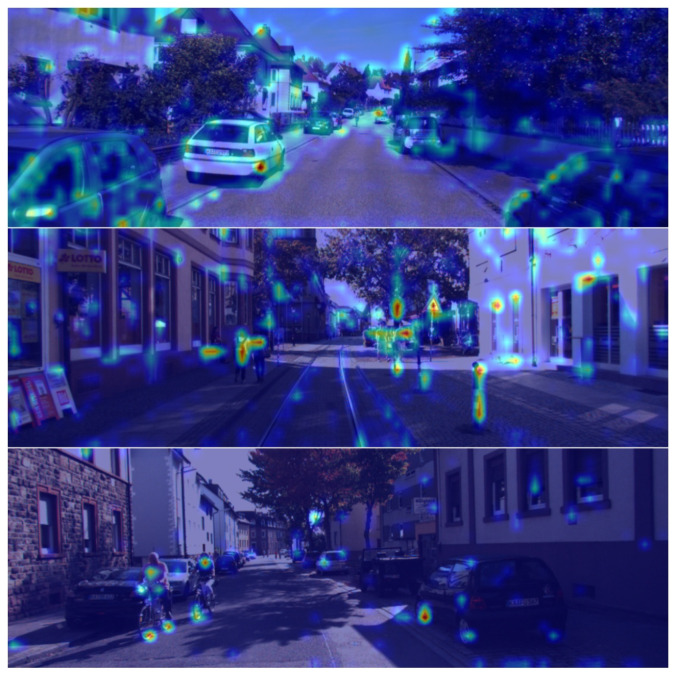
Visual representation of the BAFusion module’s attention heatmap across urban street scenes. The heatmap employs a color gradient, where warmer colors indicate higher attention weights and cooler colors denote lower attention weights. This heatmap illustrates that BAFusion preferentially allocates attention to dynamic and structurally complex objects, such as cars, cyclists, and pedestrians, rather than to static and uniform features of the environment like roads and skies. Such selective attention alignment with more challenging detection targets in point cloud data emphasizes the module’s capacity for adaptive feature utilization in multi-modal fusion for enhanced 3D object detection. Attention weights derived from BA-SECOND are applied here.

In the attention heatmap Figure 8, it is evident that BAFusion’s attention weights primarily focus on objects with distinct outlines while paying minimal attention to backgrounds such as road surfaces and skies. This may be because the image information of objects with distinct outlines can offer rich semantic information as a supplement to the corresponding point cloud features. Conversely, the image information of backgrounds such as roads and skies is less beneficial in enhancing the performance of 3D object detection. Furthermore, in scenes containing multiple object types like cars, cyclists, and pedestrians, BAFusion tends to allocate more attention to cyclists and pedestrians. This preference could be due to the fact that, compared to cars, these smaller objects have less distinct outlines in point cloud data, making the supplementation of visual information particularly crucial for their accurate identification.

This visualization provides an interpretable result of how BAFusion works, fully confirming the BAFusion module’s ability to use adaptive feature selection for multi-modal feature fusion. As a result, it facilitates the complementarity of information between point clouds and images, enhancing the overall performance of 3D object detection by leveraging the strengths of both modalities.

### 4.6. Performance Analysis of BAFusion

The experimental results and ablation studies comprehensively demonstrate the pivotal roles of the BAFusion module components in enhancing the 3D object detection capabilities of the PointPillars framework. Specifically, the Bidirectional Attention strategy further proves essential for leveraging the complementary information between LiDAR and camera data, thereby optimizing detection performance. The CFLAF layer notably enhances 3D detection accuracy and computational efficiency, proving its superiority to traditional attention methods. Moreover, the experiments validate the influence of image feature quality and position encoding on BAFusion, shedding light on the nuanced details of its fusion process.

Furthermore, attention visualization insights confirm BAFusion’s proficiency in discriminating and prioritizing salient image features, particularly enhancing the detection of smaller objects by focusing on semantic-rich outlines, further evidencing the module’s adeptness in adaptive multi-modal fusion. Moreover, the detailed comparison with SupFusion corroborates this perspective. Unlike SupFusion [56], which enhances target information in point clouds through its Polar Sample method, our model focuses on deeper attention interactions between modalities. Consequently, it achieves superior detection performance for smaller objects such as cyclists and pedestrians. These insights not only validate the proposed BAFusion’s design rationale but also offer valuable guidelines for future research in multi-modal fusion for 3D object detection.

Despite these positive outcomes, our approach encounters certain limitations that necessitate further investigation. The experimental results revealed that the addition of camera data did not always significantly enhance model accuracy, particularly in the Car category. In some cases, the performance even fell short compared to state-of-the-art point-voxel hybrid networks like PV-RCNN [37] and 3ONet [38]. These networks have been meticulously designed to exploit the unique spatial properties of point clouds in three-dimensional space, showcasing a thorough and targeted approach to feature extraction and fusion that our method has not yet fully realized.

A critical observation from our study is the relatively superficial level of feature fusion achieved. Current enhancements to the attention mechanism have primarily focused on computational efficiency rather than delving into more complex, effective fusion mechanisms. The simplistic additive approach employed in the attention fusion phase may not fully harness the rich, complementary information available from each modality. This could potentially result in a fusion that lacks depth, thereby constraining the expressiveness and performance of the derived features. Additionally, BEVFusion [55] failed to demonstrate its best performance in single-view scenarios, which has led us to realize that integrating information from multi-view images could likely yield a significant improvement in performance, a factor that has not yet been considered in our current approach.

Furthermore, although our model can be seamlessly integrated into pure point-based or voxel-based methods, it has not yet been adapted for use with hybrid point-voxel approaches like PV-RCNN and 3ONet. Developing a method to incorporate our fusion concept effectively within these hybrid systems represents a crucial direction for future research.

## 5. Conclusions

In this paper, we propose BAFusion, a novel approach that utilizes a cross-attention mechanism for adaptive LiDAR–Camera information fusion. Unlike conventional methods, BAFusion enables a soft association between images and point clouds, thereby eliminating the need for projection matrices to achieve rigid alignment between these modalities. This innovative fusion technique significantly enhances 3D object detection by effectively leveraging the complementary strengths of LiDAR and camera data, while maintaining a balance between performance enhancement and computational efficiency.

The comprehensive experiments provide profound insights into the impact of various components. These experiments not only validate the effectiveness of BAFusion but also contribute to a deeper understanding of how adaptive fusion mechanisms can be optimized for 3D object detection. However, the performance gains from BAFusion are not consistent across all scenarios. This inconsistency highlights a critical area for improvement: while BAFusion enhances computational efficiency and maintains overall detection accuracy, its current fusion mechanism does not always effectively capture the rich, complementary details of each modality. This observation underscores the need for developing more sophisticated fusion strategies that can better handle the complex interactions of multi-modal data to fully exploit their potential in enhancing detection performance.

Looking forward, we envision several promising directions to further enhance the capabilities of BAFusion. First, integrating additional sensory modalities, such as radar and infrared sensors, could provide richer contextual information, enabling more robust and accurate detections under varied environmental conditions. Second, refining the attention mechanism to incorporate dynamic weighting and context-aware strategies could address the current limitations in handling complex multi-modal interactions, potentially leading to a deeper and more nuanced fusion process. Furthermore, designing methods to incorporate multi-view image data into the fusion process could provide the network with richer environmental perception information, thereby further enhancing detection performance. Additionally, exploring real-time deployment scenarios and developing optimized models for edge computing devices will be crucial to ensure that BAFusion can be effectively utilized in autonomous systems operating in the field.

In conclusion, the promising results achieved with BAFusion highlight the potential of innovative fusion methods in advancing the perception capabilities of robotics and autonomous driving technologies.

## Figures and Tables

**Table 1:** The improvement in 3D performance by adding the BAFusion module to different point cloud baseline networks on the KITTI test set. The results are reported in Average Precision (AP) (%). Methods marked with an asterisk (*) were reproduced using MMDetection3D [48]. The best results are highlighted in bold.

Network	Cars AP (%)			Cyclist AP (%)			Pedestrian AP (%)		
	Easy	Mod.	Hard	Easy	Mod.	Hard	Easy	Mod.	Hard
PointPillars *	81.50	71.97	65.44	69.76	54.04	46.39	40.24	33.43	30.16
+ BAFusion	**82.47**	**72.21**	**67.30**	**71.94**	**56.27**	**48.63**	**46.28**	**37.57**	**34.20**
SECOND *	82.02	**72.68**	66.27	73.41	57.81	51.68	44.12	35.19	32.02
+ BAFusion	**82.62**	72.46	**67.26**	**77.31**	**61.06**	**55.07**	**50.39**	**39.98**	**35.98**
Part-A^2^ *	86.82	76.09	72.74	76.92	61.83	55.51	44.67	36.84	34.48
+ BAFusion	**86.97**	**76.31**	**73.03**	**79.31**	**63.07**	**57.44**	**47.67**	**39.21**	**36.53**

**Table 2:** The improvement in BEV performance by adding the BAFusion module to different point cloud baseline networks on the KITTI test set. The results are reported in AP (%). Methods marked with an asterisk (*) were reproduced using MMDetection3D [48]. The best results are highlighted in bold.

Network	Cars AP (%)			Cyclist AP (%)			Pedestrian AP (%)		
	Easy	Mod.	Hard	Easy	Mod.	Hard	Easy	Mod.	Hard
PointPillars *	88.39	85.57	78.66	75.76	**61.70**	54.75	45.74	38.07	35.75
+ BAFusion	**91.07**	**85.79**	**80.74**	**77.24**	61.34	**54.97**	**53.16**	**43.96**	**41.21**
SECOND *	90.52	**86.39**	**81.49**	77.98	62.61	55.67	49.46	40.22	37.46
+ BAFusion	**91.15**	85.83	81.18	**81.99**	**65.62**	**59.19**	**54.33**	**44.64**	**40.68**
Part-A^2^ *	91.75	87.51	83.01	82.10	**67.12**	60.42	48.45	41.24	38.92
+ BAFusion	**91.96**	**87.89**	**83.31**	**82.22**	67.05	**61.08**	**53.05**	**44.18**	**41.54**

**Table 3:** Comparison of 3D object detection performance for various methods on the KITTI test set, presented as Average Precision (AP) for 3D boxes. Data are sourced directly from respective publications or the official KITTI leaderboard, ensuring a reliable comparison. The best results are highlighted in bold. (L: LiDAR-based, F: LiDAR–Camera fusion).

Method	Cars AP (%)			Cyclist AP (%)		
	Easy	Mod.	Hard	Easy	Mod.	Hard
MV3D [13] (F)	74.97	63.63	54.00	N/A	N/A	N/A
VoxelNet [36] (L)	77.47	65.11	57.73	61.22	48.36	44.37
AVOD [14] (F)	76.39	66.47	60.23	57.19	42.08	38.29
F-PointNet [39] (F)	82.19	69.79	60.59	72.27	56.12	49.01
EPNet [12] (F)	89.81	79.28	74.59	N/A	N/A	N/A
PointPainting [7] (F)	82.11	71.70	67.08	77.63	**63.78**	55.89
PI-RCNN [52] (F)	84.37	74.82	70.03	N/A	N/A	N/A
TANet [53] (L)	84.39	75.94	68.82	75.70	59.44	52.53
H^2^3D RCNN [54] (L)	**90.43**	**81.55**	**77.22**	78.67	62.74	55.78
PointRCNN [35] (L)	86.96	75.64	70.70	74.96	58.82	52.53
BA-Part-A^2^ (Ours)	86.97	76.31	73.03	**79.31**	63.07	**57.44**

**Table 4:** Comparison of 3D object detection performance for various methods on the KITTI test set, presented as Average Precision (AP) for bird’s eye view. Data are sourced directly from respective publications or the official KITTI leaderboard, ensuring a reliable comparison. The best results are highlighted in bold. (L: LiDAR-based, F: LiDAR–Camera fusion, S: stereo-based).

Method	Cars AP (%)			Cyclist AP (%)		
	Easy	Mod.	Hard	Easy	Mod.	Hard
MV3D (F)	86.62	78.93	69.80	N/A	N/A	N/A
VoxelNet (L)	89.35	79.26	77.39	66.70	54.76	50.55
AVOD (F)	89.75	84.95	78.32	64.11	48.15	42.37
F-PointNet (F)	91.17	84.67	74.77	77.26	61.37	53.78
EPNet (F)	**94.22**	88.47	83.69	N/A	N/A	N/A
PointPainting (F)	92.45	88.11	83.36	**83.91**	**71.54**	**62.97**
PI-RCNN (F)	91.44	85.81	81.00	N/A	N/A	N/A
TANet (L)	91.58	86.54	81.19	79.16	63.77	56.21
H^2^3D RCNN (L)	92.85	**88.87**	**86.07**	82.76	67.90	60.49
PointRCNN (L)	86.96	75.64	70.70	74.96	58.82	52.53
BA-Part-A^2^ (Ours)	91.96	87.89	83.31	82.22	67.05	61.08

**Table 5:** The improvement in 3D performance by adding the BAFusion module to different point cloud baseline networks on the KITTI Validation set. The results are reported in Average Precision (AP) (%). The best results are highlighted in bold.

Network	Cars AP (%)			Cyclist AP (%)			Pedestrian AP (%)		
	Easy	Mod.	Hard	Easy	Mod.	Hard	Easy	Mod.	Hard
PointPillars	85.26	76.16	73.35	81.13	62.89	59.02	54.49	47.81	42.80
+ BAFusion	**88.04**	**78.77**	**74.38**	**87.81**	**65.65**	**60.96**	**61.69**	**54.83**	**49.24**
SECOND	**89.35**	79.02	74.07	77.51	62.49	58.63	59.48	52.86	47.35
+ BAFusion	89.17	**79.27**	**74.72**	**86.48**	**72.99**	**68.86**	**63.22**	**56.20**	**49.47**
Part-A^2^	91.83	82.41	80.13	88.47	70.17	66.18	62.29	54.45	48.57
+ BAFusion	**92.17**	**82.55**	**80.18**	**92.80**	**72.58**	**68.04**	**70.39**	**61.67**	**55.00**

**Table 6:** The improvement in BEV performance by adding the BAFusion module to different point cloud baseline networks on the KITTI Validation set. The results are reported in Average Precision (AP) (%). The best results are highlighted in bold.

Network	Cars AP (%)			Cyclist AP (%)			Pedestrian AP (%)		
	Easy	Mod.	Hard	Easy	Mod.	Hard	Easy	Mod.	Hard
PointPillars	92.07	88.00	83.49	83.57	66.63	62.71	60.22	53.79	48.92
+ BAFusion	**92.56**	**88.51**	**84.08**	**89.14**	**67.93**	**63.38**	**67.89**	**60.71**	**55.90**
SECOND	**93.55**	88.42	85.61	83.32	67.84	64.10	64.33	58.59	52.71
+ BAFusion	92.80	**88.43**	**85.83**	**89.40**	**77.46**	**73.11**	**66.56**	**60.85**	**54.37**
Part-A^2^	**92.95**	88.64	88.37	91.52	74.55	70.49	64.45	57.84	52.66
+ BAFusion	92.89	**88.76**	**88.39**	**93.55**	**74.82**	**70.51**	**71.70**	**64.15**	**58.56**

**Table 7:** The 3D performance comparison of BAFusion, SupFusion, and BEVFusion on the KITTI Validation set. The results are reported in Average Precision (AP) (%). The best results are highlighted in bold. Note that BEVFusion is presented separately, as it is a standalone model system, in contrast to BAFusion and SupFusion, which are enhancements designed to integrate with existing baseline networks.

Network	Cars AP (%)			Cyclist AP (%)			Pedestrian AP (%)		
	Easy	Mod.	Hard	Easy	Mod.	Hard	Easy	Mod.	Hard
BEVFusion	91.48	80.62	78.79	89.23	70.26	67.61	68.92	59.82	54.90
Sup-PointPillars	86.73	78.86	75.23	85.67	66.82	62.83	58.17	50.21	46.86
BA-PointPillars	88.04	78.77	74.38	87.81	65.65	60.96	61.69	54.83	49.24
Sup-SECOND	89.99	80.42	75.81	82.98	68.56	63.34	62.19	54.93	48.87
BA-SECOND	89.17	79.27	74.72	86.48	**72.99**	**68.86**	63.22	56.20	49.47
Sup-Part-A^2^	**92.59**	**83.23**	**80.50**	92.41	71.74	67.16	67.62	60.38	53.13
BA-Part-A^2^	92.17	82.55	80.18	**92.80**	72.58	68.04	**70.39**	**61.67**	**55.00**

**Table 8:** The BEV performance comparison of BAFusion, SupFusion, and BEVFusion on the KITTI Validation set. The results are reported in Average Precision (AP) (%). The best results are highlighted in bold. Note that BEVFusion is presented separately, as it is a standalone model system, in contrast to BAFusion and SupFusion, which are enhancements designed to integrate with existing baseline networks.

Network	Cars AP (%)			Cyclist AP (%)			Pedestrian AP (%)		
	Easy	Mod.	Hard	Easy	Mod.	Hard	Easy	Mod.	Hard
BEVFusion	91.53	88.22	87.39	91.29	74.41	72.65	70.77	**65.23**	**60.78**
Sup-PointPillars	92.30	89.76	84.10	87.67	68.33	63.89	64.50	57.80	53.10
BA-PointPillars	92.56	88.51	84.08	89.14	67.93	63.38	67.89	60.71	55.90
Sup-SECOND	**93.78**	88.59	85.95	86.72	75.05	69.20	64.36	58.93	53.01
BA-SECOND	92.80	88.43	85.83	89.40	**77.46**	**73.11**	66.56	60.85	54.37
Sup-Part-A^2^	92.35	**90.83**	**89.31**	91.34	72.97	68.48	70.46	62.96	55.09
BA-Part-A^2^	92.89	88.76	88.39	**93.55**	74.82	70.51	**71.70**	64.15	58.56

**Table 9:** Performance comparison of the BAFusion module using sequential and parallel processing methods for Car, Pedestrian, and Cyclist categories at Easy, Moderate, and Hard difficulty levels. The best results are highlighted in bold.

Method	Car AP (%)			Cyclist AP (%)			Pedestrian AP (%)		
	Easy	Mod.	Hard	Easy	Mod.	Hard	Easy	Mod.	Hard
baseline	85.26	76.16	73.35	81.13	62.89	59.02	54.49	47.81	42.80
Parallel	85.92	77.28	73.37	81.45	63.34	59.15	55.15	47.71	43.29
Sequential	**88.79**	**79.18**	**74.52**	**85.51**	**64.17**	**60.12**	**60.75**	**54.66**	**48.49**

**Table 10:** Performance comparison of the BAFusion module based on Single Attention (SA) and Bidirectional Attention (BA) for Car, Pedestrian, and Cyclist categories at Easy, Moderate, and Hard difficulty levels. The best results are highlighted in bold.

Method	Car AP (%)			Cyclist AP (%)			Pedestrian AP (%)		
	Easy	Mod.	Hard	Easy	Mod.	Hard	Easy	Mod.	Hard
baseline	85.26	76.16	73.35	81.13	62.89	59.02	54.49	47.81	42.80
SA based	87.82	**79.55**	74.31	83.34	63.31	59.79	58.45	51.46	46.55
BA based	**88.79**	79.18	**74.52**	**85.51**	**64.17**	**60.12**	**60.75**	**54.66**	**48.49**

**Table 11:** Three-dimensional performance comparison of the BAFusion module based on Conventional Attention (CA), Efficient Attention (EA), and Focused Linear Attention (FLA) for Car, Pedestrian, and Cyclist categories at Easy, Moderate, and Hard difficulty levels. The best results are highlighted in bold.

Method	Car AP (%)			Cyclist AP (%)			Pedestrian AP (%)		
	Easy	Mod.	Hard	Easy	Mod.	Hard	Easy	Mod.	Hard
baseline	85.26	76.16	73.35	81.13	62.89	59.02	54.49	47.81	42.80
CA based	88.64	**80.03**	**74.79**	84.37	**64.52**	**61.26**	58.66	52.62	47.94
EA based	85.92	77.28	73.37	81.45	63.34	59.15	55.15	47.71	43.29
FLA based	**88.79**	79.18	74.52	**85.51**	64.17	60.12	**60.75**	**54.66**	**48.49**

**Table 12:** Efficiency metrics comparison of BAFusion based on FLA and Conventional Attention.

Method	Speed (FPS)	Memory (MB)	Size (MB)
baseline	63.4	250	61
based on CA	13.9	5025	185
based on FLA (BA)	26.8	650	115
based on FLA (SA)	32.5	630	90

**Table 13:** Three-dimensional object detection performance comparison across different backbones and pre-training conditions for BA-PointPillars. The best results are highlighted in bold.

Method	Car AP (%)			Cyclist AP (%)			Pedestrian AP (%)		
	Easy	Mod.	Hard	Easy	Mod.	Hard	Easy	Mod.	Hard
CSPNet	88.47	79.00	74.39	83.12	63.48	59.45	54.43	48.73	44.13
+pre-trained	**88.79**	**79.18**	**74.52**	**85.51**	**64.17**	**60.12**	**60.75**	**54.66**	**48.49**
ResNet	**88.57**	78.76	74.15	84.61	63.24	59.11	60.74	54.15	48.92
+pre-trained	88.04	**78.77**	**74.39**	**87.81**	**65.65**	**60.96**	**61.69**	**54.83**	**49.24**

**Table 14:** Three-dimensional object detection performance comparison across different position encoding for BA-PointPillars. The best results are highlighted in bold.

PE	Car AP (%)			Cyclist AP (%)			Pedestrian AP (%)		
	Easy	Mod.	Hard	Easy	Mod.	Hard	Easy	Mod.	Hard
Fixed	88.08	79.08	73.62	82.76	62.72	58.49	57.67	53.10	46.77
Learnable	**89.23**	**80.07**	73.63	84.75	63.46	57.67	58.93	53.01	**48.78**
No PE	88.79	79.18	**74.52**	**85.51**	**64.17**	**60.12**	**60.75**	**54.66**	48.49

## Data Availability

The data supporting the findings of this study are available within the article. The experiments conducted in this research are based on the KITTI dataset, which is a publicly available benchmark dataset for autonomous driving scenarios. The KITTI dataset can be accessed at http://www.cvlibs.net/datasets/kitti/ (accessed on 24 March 2024).
